# A Comprehensive Review on a Prosthetic Finger Fabrication Approach: Clinical Rest Position (CRP) vs Palms and Fingers Extended (PFE)

**DOI:** 10.7759/cureus.67035

**Published:** 2024-08-16

**Authors:** Arunoday Kumar, Thingujam Debica, Manjula Das, Khumanthem Savana, Bankim Ningthoujam, Wahengbam Malvika

**Affiliations:** 1 Department of Prosthodontics and Crown and Bridge, Dental College, Regional Institute of Medical Sciences (RIMS), Imphal, IND; 2 Department of Prosthodontics, Government Dental College, Silchar, Silchar, IND; 3 Department of Orthodontics and Dentofacial Orthopedics, Dental College, Regional Institute of Medical Sciences (RIMS), Imphal, IND; 4 Department of Oral and Maxillofacial Surgery, Dental College, Regional Institute of Medical Sciences (RIMS), Imphal, IND

**Keywords:** clinical rest position (crp), palms and fingers extended (pfe), congenital abnormalities, finger loss, prosthetic rehabilitation

## Abstract

Creating effective prosthetic fingers involves precise treatment planning and skilled fabrication to restore aesthetic appearance and passive function. Successful outcomes depend significantly on retention and how closely the prosthesis mimics natural finger contours. We analyzed techniques for fabricating finger prostheses with palms and fingers in clinical rest position (CRP) versus palms and fingers extended (PFE) straight out. The aesthetics and function (passive) were also examined when fabricated in these two physiological conditions. We reviewed 20 articles from national and international journals over 20 years. Most literature focuses on extended posture fabrication, with few addressing CRP. This review article compares the CRP and PFE prosthetic finger fabrication approaches and explains how a prosthetic finger fabricated in a CRP offers superior aesthetic and partial functional outcomes and emerges as a promising alternative to the PFE. The potential impact of these findings on prosthodontics is significant, emphasizing the need for further research to validate these results and the ongoing development in the field.

## Introduction and background

Fingers are critical in everyday tasks, from grasping objects to intricate manipulations that define our daily lives. Fingers help us grasp objects, perform our daily functions, and give us an aesthetic appearance [1]. The finger is divided into three segments: distal phalanx, middle phalanx, and proximal phalanx. The distal and proximal phalanx are towards each finger's tip and body.

Loss of a finger, whether congenital or acquired, impacts both function and appearance, necessitating prosthetic rehabilitation. Loss of a complete finger or phalanx of a finger could be caused by congenital reasons or acquired [2]. Loss of a finger for congenital reasons is due to the absence of a gene responsible for its development. Acquired reasons for the loss of digits or fingers could be because of trauma (accidental loss) or surgical excision. Loss of a finger leads to a complete or partial loss of function and aesthetics. At the same time, the patient has to face mental trauma as there is unwanted attraction by others [2]. Achieving a prosthetic finger miming natural contours and function is a significant challenge for prosthodontists. It is also challenging for the operator to make the prosthesis regain a partial day-to-day function (holding glasses, writing, etc.).

Prosthetic finger fabrication represents a multidimensional endeavor encompassing meticulous treatment planning, precise execution of prosthetic design, and the selection of appropriate materials that balance durability and aesthetic fidelity. One crucial aspect often overlooked in this process is the hand's posture clinical rest position (CRP) and palms and fingers extended (PFE) while fabricating these prostheses. Traditionally, prosthetic fingers are designed and fabricated in a PFE straight-out position, mimicking a static posture that does not necessarily reflect the hand's natural resting position. However, recent attention has turned towards exploring an alternative approach: fabricating prosthetic fingers in a CRP. This position acknowledges the natural curvature of the palm and fingers at rest, potentially offering prosthetic solutions that look more natural and intuitively function in everyday activities. This comprehensive review aims to critically examine and compare the outcomes of prosthetic finger fabrication in these two distinct hand postures: PFE and CRP. It compares some literature with different approaches to provide insights that can inform future advancements in prosthetic finger design and enhance the quality of life for individuals requiring prosthetic rehabilitation.

Problem statement for its fabrication

Fabricating prosthetic fingers involves challenges such as cost, durability, retention, and posture considerations. Most literature focuses on materials and retention methods, with limited discussion on the posture of fingers and palms during fabrication. Expensive fabrication, significant wear and tear, loss of material, discoloration, prosthesis retention, and finger posture to be considered in its fabrication have been addressed in the literature [2-5]. Numerous articles are found in the literature elaborating on the material used in its fabrication in terms of cost and durability for their wear and tear and color stability [6-8]. Many articles are also found in the literature on increasing the retention of the prosthesis with various mechanical tools [1,2,9]. However, very few articles in the literature focus on the posture of the finger to be considered, i.e., CRP and PFE [2]. This article compares the finger prosthesis when fabricated in CRP versus PFE straight out in terms of its passive functionality and the aesthetic achieved after fabrication.

Methodology

We reviewed 20 case reports detailing techniques for fabricating finger prostheses. The articles were analyzed for aesthetics and functionality in different finger and palm postures: clinical rest and extended straight out. The procedure involved in its fabrication was studied, and the precision and perfection of the prosthesis were considered to evaluate its aesthetics and functionality in two different clinical postures of the prosthetic fingers and palms, i.e., CRP and PFE (straight out). 

Published articles, from the year 2008 to 2024, on finger prostheses were collected from online databases of PubMed, Sage journals, DOAJ, Wikipedia, Science Direct, and Google Scholar. Articles published in various national and internationally indexed journals were included in this literature review to ensure up-to-date information and technological advancements. The articles from non-peer-reviewed journals, opinion pieces, or non-scientific literature and studies that do not specifically address prosthetic finger fabrication were excluded from this literature review.

## Review

Prosthetic finger fabrication is pivotal in restoring functional capability and aesthetic appearance for individuals with finger loss or congenital abnormalities. Two primary methods, CRP and PFE, have emerged as key approaches in prosthetic finger design and fabrication. This literature survey aims to comprehensively review and compare these two techniques based on existing research and clinical evidence. It compares two fabrication approaches, CRP and PFE, offering insights into their respective aim, functionality, merits, and demerits in prosthetic finger fabrication, as given in Table 1.

**Table 1:** Comparative analysis of the fabrication approaches (CRP and PFE) CRP, Clinical rest position; PFE, Palms and fingers extended

Fabrication method	Aim of approach	Position of fingers	Functionality	Merits	Demerits
CRP	To mimic the natural posture of the hand	Natural resting position, typically with the fingers flexed	It can enhance fine motor control for everyday activities like writing, holding glasses, etc.	More comfort, reducing fatigue during prolonged wear, natural hand alignment with residual limb anatomy, natural aesthetics	May not fully accommodate the extended position of the hand during activities requiring full hand extension. This limitation can affect functionality and usability in tasks such as grasping large objects or reaching for items placed at a distance.
PFE straight out	Aims to maximize the range of motion and functional capabilities of the prosthetic finger	Positioning the hand with the fingers and palm fully extended.	Activities requiring full hand extension, such as grasping objects at various heights or performing tasks that involve precise finger movements	Beneficial for individuals engaged in occupations or hobbies that demand extensive use of the hands and fingers in extended positions	A comfortable fit is challenging as it may not align perfectly with the residual limb's natural posture during rest.

Table 2 compares references regarding retention aids and materials, particularly silicone and acrylic resin, fabrication method, procedure, and merits.

**Table 2:** Literature review for finger prosthesis

[Ref] Author, Year, Month	Type of amputated finger	Fabrication method	Method used	Merits
[1] Nimonkar et al., 2024, Jan	Partially amputated left ring finger.	PFE using Silicone	A ring is used to enhance the retention of the prosthesis with reduced weight	Increased retention and reduced weight
[2] Kumar et al., 2023, July	Partially amputated right middle finger	CRP using	Heat cure acrylic as a maxillofacial material for finger prosthesis. Finger-ring attached to increase the retention	Optimal fit, comfortable, economical, regains the passive functionality and aesthetic greatly, increased retention, and reduced weight
[3] Bashir et al., 2022, July	Forefinger prosthesis	PFE using Silicone	Technique modified for the fabrication of silicone finger prosthesis	Less weight and good color match with silicone as a maxillofacial material, good retention
[5] Aduayom-Ahego et al., 2020, Aug	Multiple-digits loss	PFE using Silicone	Explains the technique involved in rehabilitating a patient with multiple finger loss	Real silicone maxillofacial finger prostheses regained aesthetics, brought back confidence, and became socially well-accepted
[6] Gaikwad et al., 2019, Jan-June	Amputated thumb	PFE using Silicone	Technique involved in prosthetic rehabilitation of amputated thumb with silicone prosthesis	Silicone prosthesis improves the aesthetics and passive functionality
[7] Kuret et al., 2019, June	Upper limb	PFE using Silicone	To assess the adaptability to the silicone finger prosthesis, used in patients after finger amputation	Good adaptation, aesthetics, patient satisfaction, and good results enhance the quality of life
[8] Kuret et al., 2018, Aug	42 adult patients with partial or complete single- or multiple-digit amputation of one hand	PFE using Silicone	To describe the effect of prosthetic silicone finger prostheses on passive function and holding objects	Silicone finger prostheses show slight improvement in their functionality, followed by a reduction in the grip power at their tip
[9] Thomas et al., 2017, April-June	Amputated right-hand index finger due to an industrial accident	PFE using Silicone	Fabrication of Customized abutment for an Osseointegrated supported finger prosthesis	Better retention and improved function and aesthetics
[10] Yadav et al., 2016, June	Single-finger amputation	PFE using Silicone	Fabricating ring-retained silicone finger prosthesis as a prosthetic substitute for its loss	Successful with a good prognosis
[11] Asnani et al., 2015, Jan-June	Amputated thumb	PFE using Silicone	Procedure involved in making a prosthetic silicon finger because of its accidental loss	Good aesthetic outcome, improved passive function and is comfortable
[12] Goyal et al., 2015, April	Left-hand ring finger amputation	CRP using Silicone	Recording the impression and making the finger prosthesis in a relaxed position wherein the finger joints are slightly flexed	Easy to fabricate with good aesthetic outcome and enhanced retention
[13] Kiara et al., 2015, Oct-Dec	Left-hand middle finger amputation	CRP using Silicone	Silicone rubber finger prosthesis in normal position without stretching	Silicone material provided good aesthetic and function
[14] Ahmad et al., 2014, Dec	Amputated Index Finger	PFE using Silicone	Copper wire is used to make a finger ring, enhancing the finger prosthesis's retention	Good retention and restores the aesthetics and functionality to a great extent
[15] Raghu et al., 2013, Aug	Amputated finger stump	PFE using Silicone	Enhancement of retention and comfort of finger prosthesis by modification of amputated finger stump	Good prognosis in terms of aesthetics
[16] Pattanaik et al., 2013, Dec	Left-hand little finger amputation	CRP using Silicone	Explains a customized prosthetic finger made with silicone material with a simple hairpin attachment incorporated in the distal interphalangeal joint	Attachment helps in the partial restoration of function, thereby improving the functional disability
[17] Goiato et al., 2013, Aug	Traumatic amputation of the right index finger	PFE using Silicone	The finger prosthesis is retained with the incorporated O-Ring retention system and a metallic capsule adapted to the acrylic resin	Encouraged patient to return to social life
[18] Tripathi et al., 2012, Dec	Partial loss of the right-hand fingers due to road traffic accident	PFE using Silicone	The simple technique makes a precise impression of an amputated finger devoid of air-filled spaces	This technique minimized the chances of incorporating voids, as equal pressure could be employed during the insertion of the impression cap into the defective finger
[19] Jacob et al., 2012, Aug	Partially traumatically amputated finger	PFE using Silicone	The use of suction and vacuum effect around the scored stumps aided in the retention of the finger prosthesis and was successful	Increased the retention of the prosthesis
[20] Shanmuganathan et al., 2011, Dec	Partially amputated finger left middle finger	PFE using Silicone	Silicone is a material of choice for an aesthetic finger prosthesis	The patient very well accepted prosthetic rehabilitation of lost fingers with rubber silicone material
[21] Doppen et al., 2009, Feb	Five finger amputations	PFE using Silicone	Treated the amputations with osseointegrated implants to retain the finger prosthesis	Satisfactory outcomes, both esthetically and functionally, with good osseoperception
[22] Goiato et al., 2009, Oct	Amputated right index finger	PFE using Silastic - MDX 44210 silicone material	Implant-retained finger prosthesis with a modified base of the retention system	Regained the psychological, functional, and natural appearance

Comparative studies have evaluated the effectiveness of CRP versus PFE fabrication methods in clinical settings. Research suggests that choosing these methods often depends on the individual's needs, occupational requirements, and personal preferences. The CRP fabrication technique provides a significant outcome regarding comfort, functionality, durability, and aesthetic appeal. 

The comparison reveals prostheses fabricated in clinical rest position mimic natural finger contours more accurately, enhancing aesthetics and function. Despite its potential benefits, this posture needs to be more represented in literature [1-20]. It is observed that most of the papers focus on the retention of the prosthesis and the materials used for its fabrication [1,15,19]. To enhance the retention of the prosthesis, various retentive aids were used, including osseointegrated implants. Doppen et al. [21] and Goiato et al. [22] described the techniques involved in treating patients with five-finger amputations with osseointegrated implants to retain the finger prosthesis. Materials used in its fabrication included clinical silicone and heat cure acrylic resin as the material of choice with characterized intrinsic and extrinsic colors incorporated, which would match the adjacent skin color [2,5-8,10-15,17]. Caldeira et al. reviewed using silicone material for finger prostheses in amputee patients. They inferred that there were noticeable improvements in aesthetics, passive function, and restoration of self-esteem, which changed their quality of life [4].

**Figure 1:**
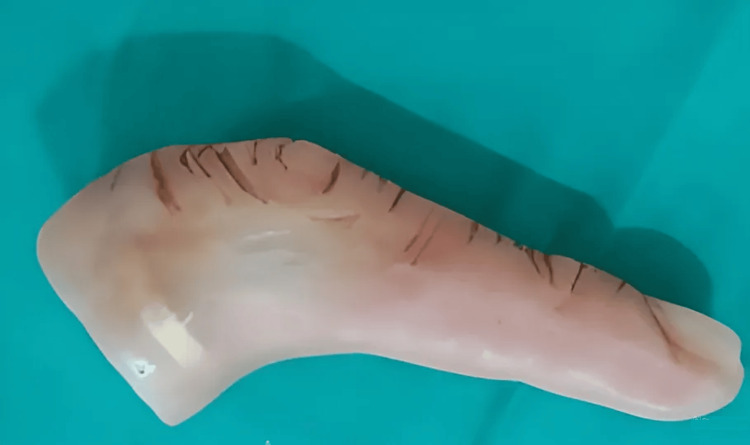
Prosthetic finger in a physiological rest position (slightly curved) made by heat cure acrylic resin with a retentive finger ring incorporated Source: Ref. [2]

**Figure 2:**
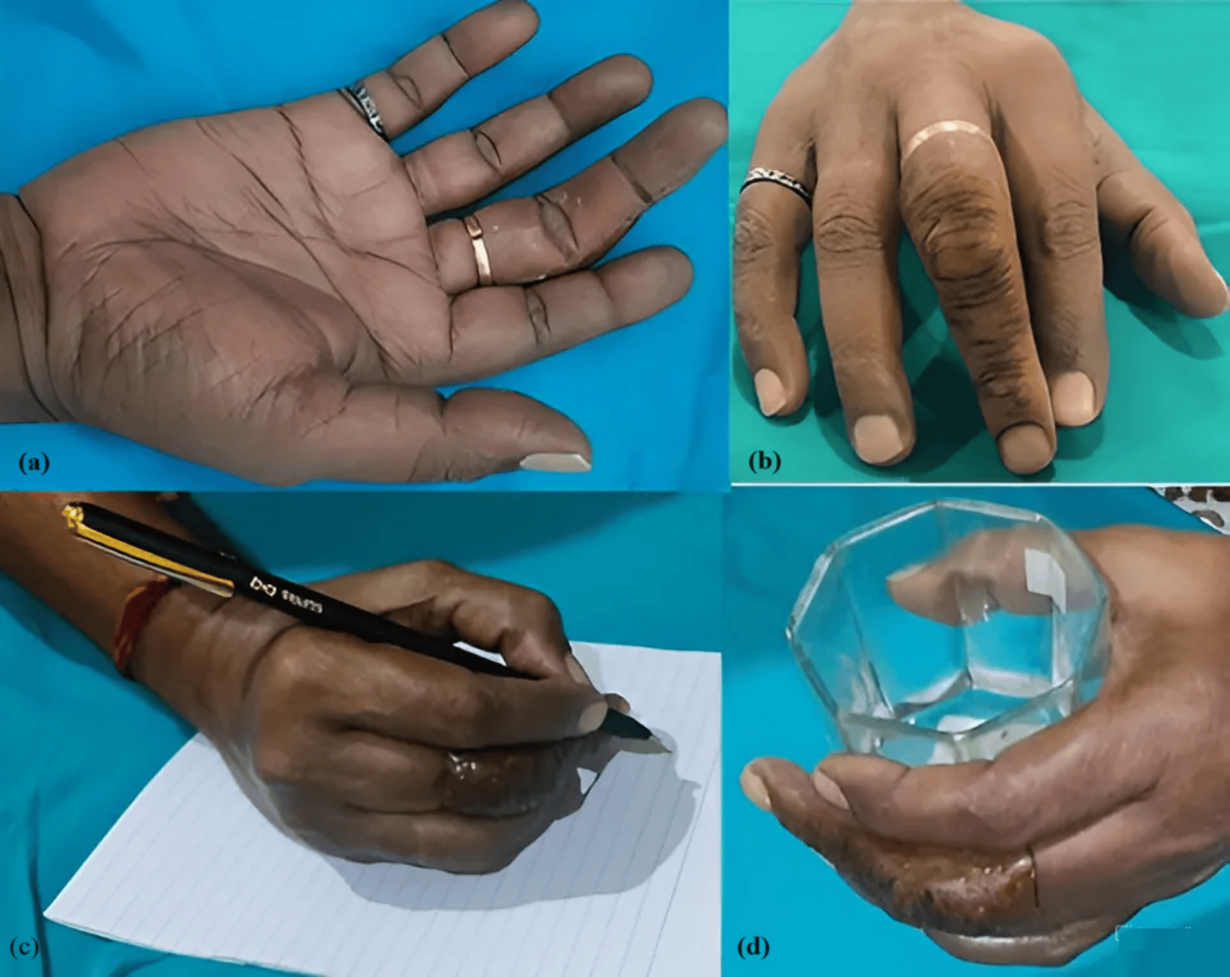
Prosthetic finger in clinical rest position (CRP) (a) Plantar surface, (b) dorsal surface, (c) writing, and (d) holding an object. Source: Ref. [2]

The posture of the palms and fingers was studied for its fabrication, from which it was inferred that none of the published articles focussed on the posture of the palms and fingers to be considered for its prosthetic fabrication. However, one article by Kumar et al. in 2023 describes a simple method to fabricate a partially amputated prosthetic finger in a physiological rest position (palms and fingers are slightly curved, and so is the prosthetic finger) with heat cure acrylic resin as a maxillofacial material of choice [2]. As documented by Kumar et al. [2], the procedure involved in its fabrication provided an aesthetic and economical way of prosthetically rehabilitating an amputated finger, which regains back the aesthetics and passive functionality to a great extent (Figures 1-2).

Meanwhile, prosthetic fingers, if fabricated when all the fingers and palms are extended straight out, are uncomfortable when performing day-to-day functions such as writing or holding objects.

## Conclusions

Existing literature offers comprehensive insights into materials, retention techniques, and psychological aspects of prosthetic finger rehabilitation, but the impact of hand posture during fabrication remains unexplored. This thorough review aims to fill this gap by comparing prosthetic rehabilitation of the imputed finger under two different postures: PRE and PFE. This review paper highlights the CRP as a promising approach for advanced prosthetic design and enhanced functional and aesthetic outcomes for individuals requiring finger prostheses. However, the selection of prosthetic finger fabrication methods based on CRP should be carefully tailored to meet each individual's unique needs and functional requirements. More intervention and clinical research are needed to enhance prosthetic fingers' design and fabrication processes. There is no bias in the assessment of individual studies, as stated.
